# Targeting the Tumor Immune Microenvironment Could Become a Potential Therapeutic Modality for Aggressive Pituitary Adenoma

**DOI:** 10.3390/brainsci13020164

**Published:** 2023-01-18

**Authors:** Zuocheng Yang, Xuemei Tian, Kun Yao, Yakun Yang, Linpeng Zhang, Ning Liu, Changxiang Yan, Xueling Qi, Song Han

**Affiliations:** 1 Department of Neurosurgery, Beijing Tiantan Hospital, Capital Medical University, Beijing 100070, China; 2 Department of Nursing, Sanbo Brain Hospital, Capital Medical University, Beijing 100093, China; 3 Department of Pathology, Sanbo Brain Hospital, Capital Medical University, Beijing 100093, China; 4 Department of Neurosurgery, Sanbo Brain Hospital, Capital Medical University, Beijing 100093, China

**Keywords:** aggressive pituitary adenoma, macrophage, CD8+ TILs, immune microenvironment, combined immunotherapy, temozolomide

## Abstract

Object: This study aimed to explore the relationship between the aggressiveness and immune cell infiltration in pituitary adenoma (PA) and to provide the basis for immuno-targeting therapies. Methods: One hundred and three patients with PA who underwent surgery at a single institution were retrospectively identified. The infiltration of macrophages and T-lymphocytes was quantitatively assessed. Results: The number of CD68+ macrophages was positively correlated with Knosp (*p* = 0.003) and MMP-9 expression grades (*p* = 0.00). The infiltration of CD163+ macrophages differed among Knosp (*p* = 0.022) and MMP-9 grades (*p* = 0.04). CD8+ tumor-infiltrating lymphocytes (TILs) were also positively associated with Knosp (*p* = 0.002) and MMP-9 grades (*p* = 0.01). Interestingly, MGMT expression was positively correlated with MMP-9 staining extent (*p* = 0.000). The quantities of CD8+ TILs (*p* = 0.016), CD68+ macrophages (*p* = 0.000), and CD163+ macrophages (*p* = 0.043) were negatively associated with MGMT expression levels. The number of CD68+ macrophages in the PD-L1 negative group was significantly more than that in the PD-L1 positive group (*p* = 0.01). The rate of PD-L1 positivity was positively correlated with the Ki-67 index (*p* = 0.046) and p53 expression (*p* = 0.029). Conclusion: Targeted therapy for macrophages and CD8+ TILs could be a helpful treatment in the future for aggressive PA. Anti-PD-L1 therapy may better respond to PAs with higher Ki-67 and p53 expression and more infiltrating CD68+ macrophages. Multiple treatment modalities, especially combined with immunotherapy could become a novel therapeutic strategy for aggressive PA.

## 1. Introduction

Pituitary adenoma (PA) accounts for approximately 15% of intracranial tumors and represents the second-most common primary brain tumor in humans [1]. Most PAs are non-aggressive, benign tumors that grow slowly in the sellar and suprasellar regions [2]. However, about 35% of PAs are aggressive adenomas that infiltrate the adjacent sphenoid sinus, cavernous sinus (CS), and internal carotid arteries [3]. In addition, third ventricular compression may occur, which leads to hydrocephalus [4]. Due to their invasive nature, the goal of complete removal of PA is difficult to achieve, which further increases the risk of tumor recurrence [4]. As a result, local compression symptoms and neuroendocrine symptoms caused by aggressive PAs remain after traditional treatment. Hence, a multidisciplinary approach including surgery, radiotherapy, chemotherapy, and immunotherapy is often required. Significant progress has been made recently in immunotherapy techniques, and they show great promise.

Tumor-associated macrophages (TAMs) are an important constituent of the tumor microenvironment and are polarized into classically activated (M1) and alternatively activated (M2) subtypes. The latter is characterized by CD163 positivity [5], whereas CD68+ is a pan-macrophage marker for both M1 and M2 macrophages. Some researchers have shown that M2 macrophages generally contribute to tumor initiation, progression, invasion, and angiogenesis and are correlated with poor prognosis of many cancers, including glioblastoma (GBM) [6,7]. CD8+ tumor-infiltrating lymphocytes (TILs) can initiate cytotoxic cascades, giving them antitumor functions [8]. Interestingly, PAs that invade the CS tend to have higher numbers of CD8+ lymphocytes than PAs that do not invade the CS [9]. In addition, the expression of programmed death-ligand 1 (PD-L1) is regarded as a crucial factor in predicting the effects of immune checkpoint inhibitors. PD-L1 expression positively correlates with CS invasion and increased TILs [9]. The application of anti-PD-L1 antibodies has achieved an encouraging outcome in a PA treatment of an in vitro animal study [10].

Two proteins are critical to monitor PA development. Matrix metalloproteinase (MMP)-9 encourages angiogenesis and promotes the invasion of many cancers and is the first member of the MMP family to be linked to the invasion of PA [11,12]. In a rat model, an MMP-9 inhibitor has shown promise as a treatment of prolactinoma [13]. In addition, O6-methylguanine-DNA methyltransferase (MGMT), which is located at chromosome 10q26, encodes a DNA repair enzyme [14]. Temozolomide (TMZ) is an alkylating chemotherapeutic agent that has been used against aggressive PA and carcinoma [15]. The presence of MGMT inhibits TMZ effectiveness, suggesting that negative MGMT expression could predict responsiveness to therapy [16]. However, the association between MGMT expression and the aggressiveness of PA remains controversial [17,18]. Consequently, it is necessary to understand comprehensively the effect of the immune microenvironment on tumor invasion to develop complete multidisciplinary treatments. The assessment of imaging for aggressive PA can help in this determination.

In this study, we primarily examined the expression of the extent of immune cell infiltration, immune checkpoint, MMP-9, and MGMT expression to investigate the relationship between these biomarkers and invasion and prognosis of PA.

## 2. Methods

### 2.1. Patients and Design

This was a retrospective study of 103 patients with PA who received surgery in SanBo Brain Hospital Capital Medical University between October 2017 and April 2018. The medical records, neuroimaging data, neuropathological material, and other data were extracted and collated from the medical databases of the hospital. There were 54 males and 49 females, and the mean age of the patients in the study was 45.02 ± 14.91 years (range, 15 to 79 years).

There were 79 patients with primary PAs and 24 patients with recurrent PAs. The PA volume was estimated from T1-enhanced magnetic resonance imaging (MRI) scans using a previously reported method [19]. The median tumor volume was 7.08 cm^3^ (range, 0.28 to 245 cm^3^). Ten patients with pituitary apoplexy were mainly diagnosed by MRI and clinical signs/symptoms [20]. The Knosp classification grade was judged based on the neuroimaging diagnostic criteria [21]. Aggressive and non-aggressive PAs represented 47.57% (grade 0 in 43 patients, grade 1 in 6 patients, and grade 2 in 10 patients) and 52.43% (grade 3 in 10 patients and grade 4 in 34 patients), respectively. According to the 2017 WHO histopathological classification [22], PAs in this study were divided into seven groups: eight cases of somatotroph, 18 cases of lactotroph, one case of thyrotroph, five cases of corticotroph, 22 cases of gonadotroph, 45 cases of null cell, and four cases of plurihormonal adenomas. In addition, secretory PAs included four growth hormone (GH)-secreting adenomas, 26 prolactinomas, eight adrenocorticotropic hormone (ACTH)-secreting adenomas, two follicle-stimulating hormone (FSH)-secreting adenomas, and 14 plus hormonal secreting adenomas; the others were non-functional adenomas (49/103, 47.57%). The detailed patient information is shown in Table 1.

### 2.2. Immunohistochemistry

Immunohistochemical studies were performed on paraffin sections using the EnVision method. The fresh PA specimens were fixed with 10% neutral buffered formalin and embedded in paraffin. Formalin-fixed and paraffin-embedded tumor specimens were cut into 4-µm thick sequential sections and processed for immunohistochemistry. The sections were then treated with 3% H_2_O_2_ for 5 min at room temperature to block endogenous peroxidase activity. The slides were blocked with 5% fetal bovine serum (Jackson ImmunoResearch Laboratories, Inc., West Grove, PA, USA) diluted in wash buffer with 1% bovine serum albumin (Merk KGaA) at room temperature for 15 min and incubated with primary antibodies against epithelial membrane or cytoplasmic antigens at 4 °C for 15 h: CD8 (1:150 dilution; mouse. clone OTI3H6; ZSGB-BIO), CD68 (1:150 dilution; mouse; clone OTI4G1; ZSGB-BIO), CD163 (1:150 dilution; mouse; clone OTI2G12; ZSGB-BIO), MMP-9 (1:200 dilution; mouse; clone 4A3; ZSGB-BIO), PD-L1 (1:500 dilution; mouse; clone OTI9E12; ZSGB-BIO), GH (1:50 dilution; rabbit; clone EP267; ZSGB-BIO), Prolactin (1:50 dilution; rabbit; clone EP193; ZSGB-BIO), TSH (1:50 dilution; rabbit; clone EP254; ZSGB-BIO), hCG (1:50 dilution; rabbit; clone 2F12; ZSGB-BIO), β-LH (1:50 dilution; rabbit; clone 2G10; ZSGB-BIO), β-FSH (1:50 dilution; rabbit; clone 2E4; ZSGB-BIO), ACTH (1:50 dilution; rabbit; clone 2F6; ZSGB-BIO), nuclear antigen Ki-67 (20 µg/mL dilution; mouse; clone 20Raj1; invitrogen), MGMT (1:500 dilution; mouse; clone OTI1B12; ZSGB-BIO), p53 (1:500 dilution; mouse; clone Pab 240; ZSGB-BIO),T-PIT (1:50 dilution; rabbit; clone OTI2G1; ZSGB-BIO), PIT-1 (1:50 dilution; rabbit; clone G-2; ZSGB-BIO), or SF-1 (1:50 dilution; rabbit; clone OTI1H2; ZSGB-BIO). After washing with phosphate-buffered saline three times, the sections were incubated with horseradish peroxidase-conjugated anti-mouse or rabbit secondary antibodies (cat. no. PV-6000D; Zhongshan Bio-Tech Co., Ltd., Zhongshan, China) for 30 min at 37 °C. Then, 3,3′-diaminobenzidine (DAB) was applied for color development at room temperature for 5 min, and sections were subsequently counterstained with hematoxylin. Samples were mounted using Faramount mounting solution (Agilent Technologies Inc., Santa Clara, CA, USA), and each slide was individually reviewed and scored by three pathologists. Ki-67 positivity was used to determine the percentage of labeled nuclei in the PA specimen [22]. More than 10% of stained tumor cell nuclei were also deemed p53-positive [23].

The stained sections were viewed at low magnification to identify 5–10 “hot-spot” areas with the highest density of stained immune cells. The number of selected “hot-spots” mainly depended on the size of the specimen under 400× microscopic examination. For every region of interest, the number of stained immune cells was counted. The average of the highest three values was used as the final value. The method is a comprehensive reference to previous studies [9,24].

Positive MMP-9 expression was present in the cytoplasm. Characteristics were scored as follows: the staining extent was described as the percentage of positive cells (0, <5%; Ⅰ+, 5–25%; Ⅱ+, 26–50%; and Ⅲ+, >50% cells positive) and the staining intensity was numerically categorized (0, no staining; Ⅰ+, pale yellow; and Ⅱ+, brown). The final scores were calculated by adding the two scores and were classified according to the following standards: 0- (negative), 1–2+ (weakly positive), 3–4++ (moderately positive), and 5+++ (strongly positive) [25]. PD-L1 membrane immunostaining in over 5% of tumor cells was considered as positive expression [26]. Less than 10% of tumor cells with nuclear staining for MGMT were deemed as low MGMT expression, 10–90% of tumor nuclei positive as intermediate expression, and ≥90% of tumor nuclei with diffuse positive staining as high expression regardless of intensity [27] (Table 1). The scoring was independently performed by three pathologists.

### 2.3. Statistical Analysis

The R language and GraphPad Prism software 9.0 (GraphPad Software, San Diego, CA, USA) were used to analyze and map the data. The Student’s *t*-test was conducted on two-group comparisons. Spearman correlations and regression analyses were mainly used to analyze the relationship between the factors. For the comparison of multiple groups, a one-way ANOVA was performed. The Chi-square test was used for categorical variables, when needed. The Kaplan–Meier method was used to evaluate the overall survival (OS) and progression-free survival (PFS). Univariate and multivariate analyses were performed with the Cox proportional hazards model. A *p* value < 0.05 was deemed significant.

## 3. Result

### 3.1. Markers of Invasiveness and Aggressive Infiltration

**Figure 1:**
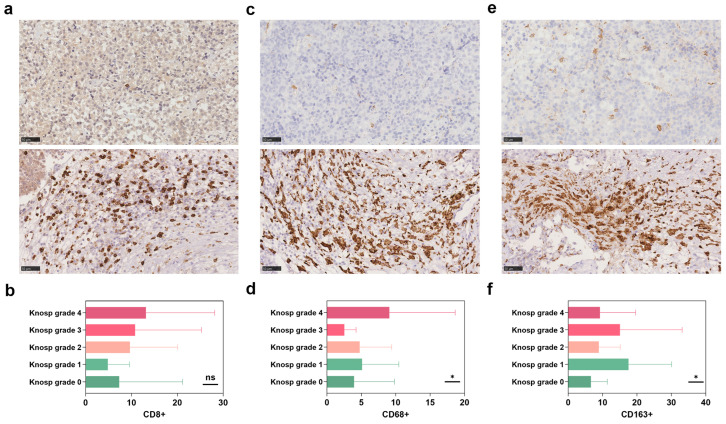
Immunohistochemistry of tissue microarray for immune cells in pituitary adenoma (PA). The different distribution of CD8+ TILs between the non-aggressive PA and aggressive PA at ×400 magnification (**a**) and the Knosp grades (**b**); the different distribution of CD68+ macrophages surrounding the vessels between the non-aggressive PA and aggressive PA at ×400 magnification (**c**) and the Knosp grades (**d**); the different distribution of the density of infiltrating CD163+ macrophages between the non- aggressive PA and aggressive PA at ×400 magnification (**e**) and the Knosp grades (**f**).

**Figure 2:**
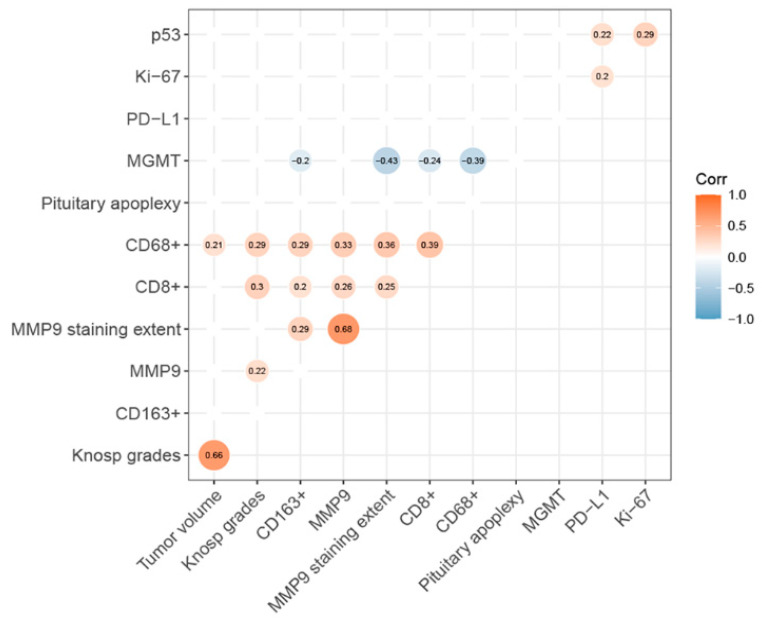
Correlation heatmap (Spearman correlation) of immune cells and pro-invasive factors. Only statistically significant differences are displayed.

CD68+ macrophage infiltration surrounding tumor blood vessels was often observed (Figure 1c). There was significant differences in the distribution of CD68+ (*p* = 0.016, Figure 1c,d) and CD163+ macrophage (*p* = 0.022, Figure 1e,f), but not in CD8+ TILs (*p* = 0.377, Figure 1a,b). Among them, post hoc analysis indicated that the number of CD163+ macrophages in tumors of Knosp grade 0 was lower than those with Knosp grade 1 and grade 3. The CD68+ cells infiltrating PAs were positively correlated with tumor volume (*p* = 0.03, r = 0.21) and Knosp grade (*p* = 0.003, r = 0.29, Figure 2). Similar to the results described above, CD8+ TILs were positively associated with Knosp grade (*p* = 0.002, r = 0.30, Figure 2).

**Figure 3:**
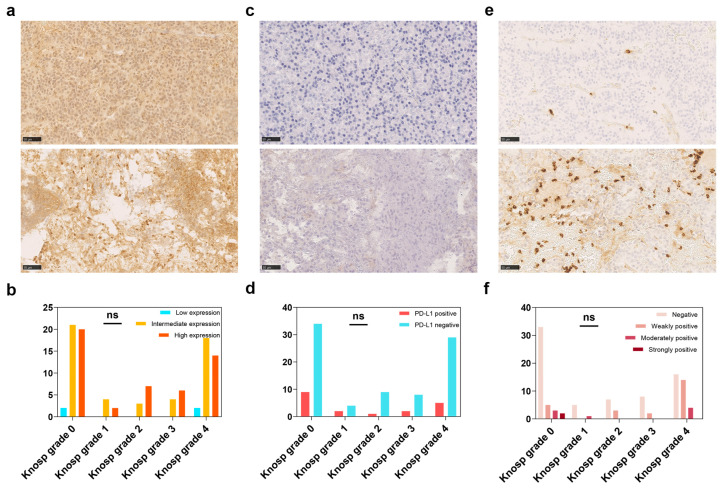
Immunohistochemical studies of O6-methylguanine-DNA methyltransferase (MGMT), PD-L1 and metalloproteinase (MMP)-9 in PA. The different MGMT expression between non-aggressive PA and aggressive PA at ×200 magnification (**a**) and the Knosp grades (**b**); the different PD-L1 expression between non-aggressive PA and aggressive PA at ×200 magnification (**c**) and the Knosp grades (**d**); the different MMP-9 expression between non-aggressive PA and aggressive PA at ×200 magnification (**e**) and the Knosp grades (**f**).

The final scores of MMP-9 staining were positively related to Knosp grade (r = 0.22, *p* = 0.03) (Figure 2); however, statistical significance was not found in the expression of MMP-9 between the Knosp grades (*p* = 0.127; Figure 3e,f). In addition, they were not related to the Ki-67 index (*p* = 0.76) and tumor volumes (*p* = 0.33). Interestingly, the final scores of MMP-9 immunoreactivity were positively correlated with the number of CD8+ TILs (r = 0.26, *p* = 0.01) and CD68+ macrophages (r = 0.32, *p* = 0.00) beyond CD163+ macrophages (*p* = 0.38). The numbers of CD163+ macrophages infiltrating PAs were highly variable with statistically significant differences among the MMP-9 scores (*p* = 0.04) and were higher in the moderately MMP-9 positive group than in the weakly positive group (*p* = 0.006).

It is interesting to note that the expression of MMP-9 was positively correlated with tumor recurrence (r = 0.23, *p* = 0.03), and patients with MMP-9 positivity had a 2.63-fold increased risk for relapse (B = 0.97, *p* = 0.03).

### 3.2. MGMT and Immune Checkpoint

There were four PAs with low MGMT expression (3.88%), 50 with intermediate (48.54%), and 49 with high MGMT expression (47.57%) on immunostaining. No significant MGMT expression between the Knosp grades was observed in this study (*p* = 0.782; Figure 3a,b).

The numbers of CD8+ TILs (r = −0.236, *p* = 0.016), CD68+ macrophages (r = −0.392, *p* = 0.000), and CD163+ macrophages (r = −0.200, *p* = 0.043) were negatively associated with MGMT expression (Figure 2). MGMT expression was not associated with Ki-67 value (*p* = 0.437), p53 expression (*p* = 0.691), and Knosp classification grade (*p* = 0.791, Figure 2 and Figure 3). Notably, MGMT expression was inversely correlated with the extent of MMP-9 staining (r = −0.442, *p* = 0.000, Figure 2).

A statistically significant difference in MGMT expression was found between primary and recurrent PAs (Chi-square = 6.646, *p* = 0.036, 5.06% vs. 0.00%). The distribution of MGMT expression did not differ among the pathological subtypes (Chi-square = 6.552, *p* = 0.886).

**Figure 4:**
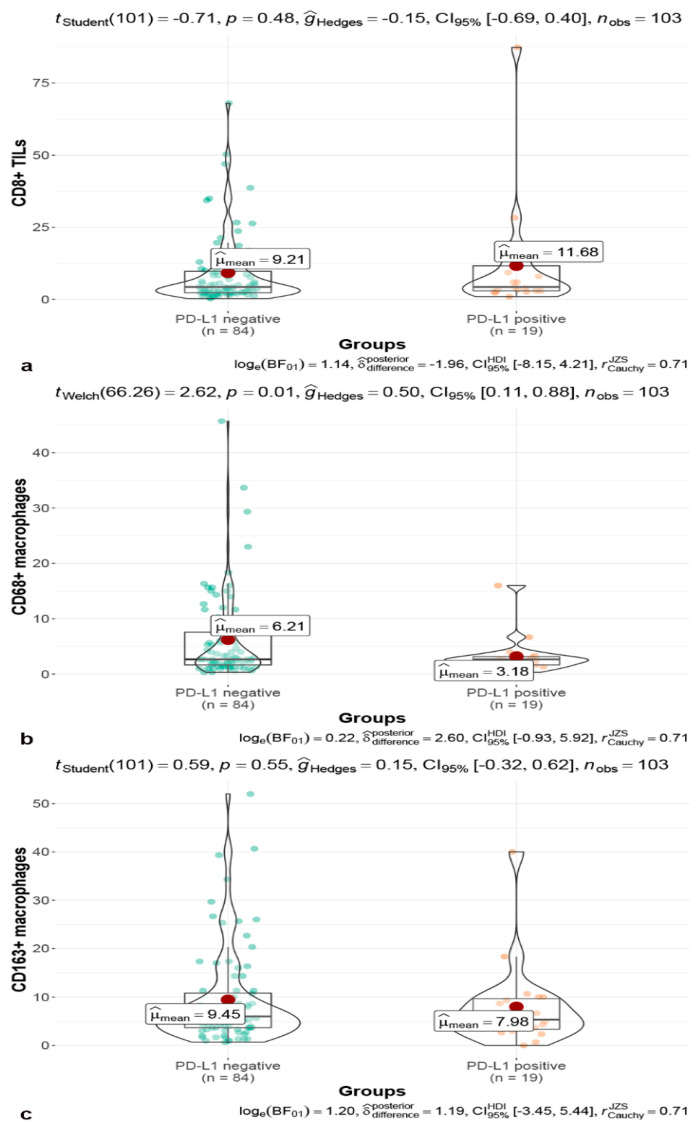
Unpaired *t*-test performed between the PD-L1 positive and PD-L1 negative groups in CD8+ TILs (**a**); the infiltration of CD68+ macrophages difference between PD-L1 positive and PD-L1 negative groups were calculated with an unpaired *t*-test (**b**); Student’s *t*-test between the PD-L1 positive and PD-L1 negative groups in CD168+ macrophages (**c**).

No significant difference was observed in the number of CD8+ TILs (*p* = 0.60, Figure 4a) and CD163+ cells (*p* = 0.53, Figure 4c) between the PD-L1 negative and positive groups. However, the number of CD68+ macrophages in the PD-L1 negative group was significantly more than that in the PD-L1 positive group (*p* = 0.01, Figure 4b). The expression of PD-L1 was not differed among the Knosp grades (*p* = 0.760; Figure 3c,d) and was associated with MMP-9 expression (*p* = 0.78, Figure 2). The expression of PD-L1 was positively correlated with Ki-67 index (*p* = 0.046, r = 0.20) and p53 expression (*p* = 0.029, r = 0.22) (Figure 2).

**Figure 5:**
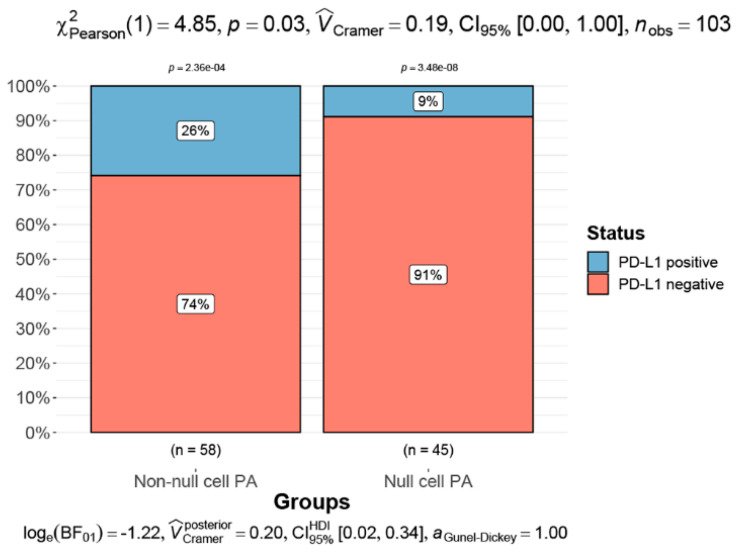
Proportions of positive PD-L1 between null cell and non-null cell groups are compared using the chi-squared test.

The rate of PD-L1 positivity was significantly higher in non-null cell PA (15/58, 25.86%) than in null cell PA (4/45, 8.89%) (Chi-square = 4.85, *p* = 0.028, Figure 5). However, a similar result was not observed in secreting PA groups (*p* = 0.122).

### 3.3. Classifications and Apoplexy of Pituitary Adenoma

There was no difference in the number of CD8+ TILs (*p* = 0.38) or CD68+ (*p* = 0.97) and CD163+ macrophages (*p* = 0.29) among the pathological classifications of tumors. The thyrotroph adenoma group was excluded since only one patient was in the group.

There was no significant difference in the distribution of CD8+ TILs (*p* = 0.691) or CD68+ (*p* = 0.074) and CD163+ macrophages (*p* = 0.123) between the non-functional and functional adenomas. The number of CD163+ macrophages differed among secreting PA groups (*p* = 0.003), and a post hoc analysis showed that the quantity of CD163+ macrophages in GH-secreting adenomas was higher than that in prolactinomas (*p* = 0.0003), ACTH-secreting adenomas (*p* = 0.007), non-functional adenomas (*p* = 0.004), and plurihormonal-secreting adenomas (*p* = 0.003). The number of non-functional adenomas was larger than prolactinomas (*p* = 0.048) and plurihormonal-secreting adenomas (*p* = 0.022)

No differences in CD8+ TILs (*p* = 0.81) or CD68+ (*p* = 0.70) and CD163+ macrophage (*p* = 0.95) distributions were seen between the pituitary apoplexy and no apoplexy group.

### 3.4. Survival Analysis

Ninety-three patients completed follow-ups, with a mean follow-up time of 30.56 ±  6.53 months. Two patients with PAs died from bleeding because of surgery complications, and two patients died of other causes. The average PFS was 29.26 ± 8.35 months (range, <1–35 months), and nine patients (9/93, 9.68%) had tumor recurrence during the follow-up. Only one patient with Knosp grade 0 received complete resection of the tumor among nine patients with tumor recurrence; seven patients with CS invasion had an incomplete tumor removal.

**Figure 6:**
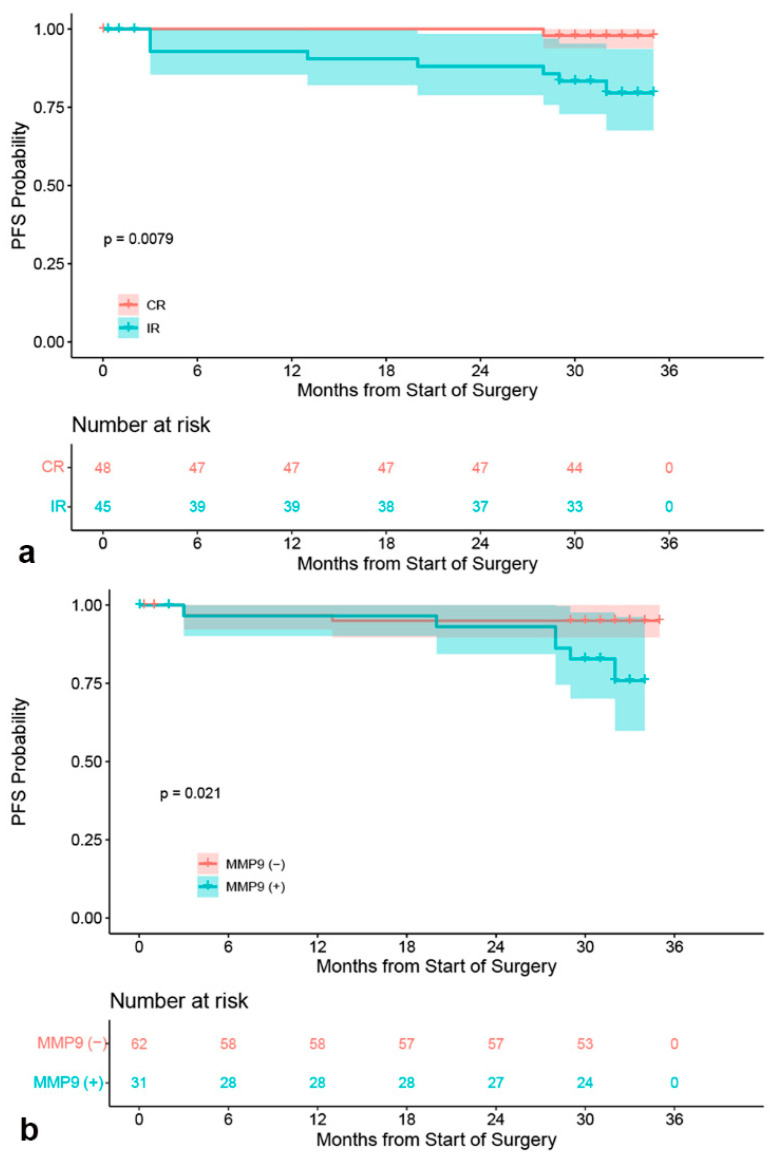
Kaplan–Meier PFS curves. PFS curve and PFS stratification based on complete resection (CR) and incomplete resection (IR) (**a**); PFS differed significantly between the MMP-9 positive and MMP-9 negative groups (**b**).

In the multivariate analyses, no independent risk factors were found for OS (Table 2). An analysis of possible risk factors for PA recurrence using multiple Cox regression showed that recurrent tumors were significantly more likely to recur (*p* = 0.04, Table 3). Univariate Cox regression analysis showed patients with MMP-9 positive expression (*p* = 0.04) and incomplete resection (*p* = 0.03) had a significantly increased risk of recurrence (Table 3). Kaplan–Meier curve analysis showed that patients with incomplete resection of the tumor had low PFS rates (*p* = 0.0079, Figure 6a). Patients with MMP-9 positive expression tended to have shorter PFS than MMP-9 negative patients (*p* = 0.021, Figure 6b).

## 4. Discussion

This study demonstrated that the number of immune cells infiltrating PAs and MGMT expression were correlated with tumor aggressiveness based on imaging and molecular features. In addition, the positive rate of PD-L1 was positively associated with the Ki-67 index and p53 expression. Therefore, the above results reveal that there will be a novel and promising therapeutic approach for the management of aggressive PA.

Our findings confirmed that infiltrating TAMs and M2 polarization in PA were correlated with Knosp grades and MMP-9 expression. However, studies investigating M2 polarized macrophages in PA are rare. Macrophages become polarized based on changes in the environment, creating the different macrophage subtypes, M1 and M2. As is well-known, M1 macrophages generally have antitumor properties and enhanced antigen-presenting ability. In contrast, M2 macrophages show reduced antimicrobial and tumoricidal activity [28]. A study by Marques et al. [29]. showed that M2 macrophages could promote neo-angiogenesis in pituitary neuroendocrine tumors (PitNET).The number of CD68+ macrophages infiltrating PAs was positively correlated with tumor size and Knosp grade, which indicates tumor aggressiveness [24]. TAMs could enwrap tumor-associated blood vessels and create an environment conducive to tumor progression by promoting tumor angiogenesis and secreting growth factors [30]. MMP-9, as a member of the zinc-dependent endopeptidase family, could promote angiogenesis and cancer invasion by degrading type IV collagen of basal membrane near the tumor cell and extracellular matrix (ECM) [31]. There are few studies on this topic, and further work is required to validate the specific mechanism.

It has been reported that the number of CD68+ macrophages in sparsely granulated GH-secreting adenomas is greater than in ACTH adenomas, and nonfunctional adenomas show more infiltrating CD68+ macrophages than ACTH adenomas [24]. Interestingly, only GH-secreting adenomas had the highest number of CD163+ macrophage infiltration, which may support the evidence that GH adenomas tend to have an aggressive behavior [32].

Controversies exist regarding the associations between CD8+ TILs and PA invasiveness or aggressiveness. It has been reported that T-lymphocytes (CD8, CD4, FOXP3) recruited by pituitary neuroendocrine tumor-derived chemokines determine the aggressive behavior [33]. A tendency for higher invasiveness or aggressiveness in PAs with more infiltration of CD8+ TILs has been observed [9,34]. However, it was noted that the number of CD8+ lymphocytes was positively correlated with Knosp grades in our study.

In addition, the number of CD8+ TILs was positively correlated with the expression of MMP-9. MMP-9 plays a critical role in CD8+ T-cell infiltration into tissues and exerts a regulatory role on CD8+ T-cell activation [35,36]. Our results revealed that PAs with aggressive behavior and/or MMP-9 expression could present a microenvironment highly infiltrated by CD8+ TILs, which may exert a specific effect on the invasion of PAs. Whether CD8+ TILs could encourage an immunosuppressive phenotype or not warrants further study.

There was a trend suggesting a higher positive rate of PD-L1 in aggressive PA and non-functional PA [9,34]. However, the present results only confirmed that a higher expression of PD-L1 occurred in null cell PAs than the other PAs. In addition, it was reported that PD-L1 expression is positively associated with increased numbers of CD8+ TILs [34]. However, this result was not seen in this study. Interestingly, CD68+ macrophages were more infiltrated in the positive PD-L1 group. This result suggests that CD68+ macrophages may promote the expression of PD-L1, further encouraging the formation of an immunosuppressive microenvironment. In addition, it seems that patients with positive p53 expression and a high Ki-67 index would benefit most from anti-PD-L1 therapy. Although some studies on glioblastomas showed a correlation between PD-L1 and MGMT expression [37,38], a similar result was not found in this study.

A previous study demonstrated that MMP-9 has potential as a marker for invasion but not for recurrence [39]. Survival analysis showed that patients with positive MMP-9 expression had worse PFS and a higher risk of tumor recurrence. In addition, incomplete resection was a significant independent predictor of recurrence of tumor in multivariate analyses, and patients with incomplete removal of the tumor had a significantly shorter PFS in this study.

The lower the MGMT expression, the better the response to the TMZ treatment in PA [40]. Our results suggested that there may be some interaction between the aggressiveness of the tumor and MGMT expression. A previous study showed that low-to-moderate MGMT immunoexpression occurred significantly more often in aggressive PAs [41]. However, although it was not significantly different between aggressive and non-aggressive PAs in the imaging data, the level of MGMT immunopositivity was positively associated with the MMP-9 staining extent. This result may further exemplify that aggressive PAs could be a suitable candidate for TMZ therapy, but it is necessary to provide more evidence to document this. In addition, the expression of MGMT did show linear relationships with infiltrating immune cells, which may provide evidence that it is possible to use immunotherapy in combination with TMZ to treat aggressive PA.

Together, our results demonstrate that the tumor immune microenvironment serves a vital role in the invasion of PA. However, the effect of immunotherapy on pituitary adenomas has only been reported in some cases, and the effect is uncertain. One patient with ACTH-secreting PA progressed rapidly after four cycles of anti-PD-L1 (pembrolizumab) [42]. Clinical trials showed that the therapeutic effect of a single immune checkpoint inhibitor is not satisfactory for GBM, and attention has shifted focus to immunotherapies combined with other treatments. This novel approach might be more beneficial for patients with GBM [43]. There are currently only two clinical trials of combined immunotherapy for pituitary tumors in clinicaltrials.gov accessed on 2 November 2022. Combined immunotherapy (anti-PD-L1 and CTLA-4) for aggressive pituitary adenomas is still in phase 2 (Memorial Sloan Kettering cancer center, United States, NCT04042753). Another clinical trial of combined immunotherapy for rare pituitary tumors is still recruiting patients (National Cancer Institute, United States, NCT02834013). At present, there are no animal experiments or clinical studies on macrophage-targeting treatment for PA. In addition, the number of cases with a low expression of MGMT is relatively small. Unfortunately, a 2017 review reports that only approximately 42% of pituitary tumors show a radiological response to TMZ treatment [44].

It was noteworthy that in 2022, the WHO emphasized the various cell types and their subtypes and classifies new subtypes of the PitNET based on the tumor cell lineage, cell type, and related characteristics. Therefore, the new classification criteria may bring variations in immune cell infiltration and expression of MGMT, MMP-9, and PD-L1 between the different categories in this study. However, despite the controversies in the field, there is no novel definition of aggressive PA/PitNET in the 2022 WHO classification of pituitary tumors. Nevertheless, some subtypes of PitNETs, such as Crooke cell and immature PIT1-lineage tumors, may have features of aggressive behaviors. It is believed that the diagnosis of “aggressive PitNETs” in the future may also be mainly based on cell type, tumor lineage, and clinical features, and the cure of the disease might benefit primarily from new molecular or immune drugs.

In summary, we confirmed that there are significant differences in the infiltration of immune cells (CD8+ TILs, CD68+ and CD163+ macrophages) and the expression of PD-L1 and MGMT between aggressive and non-aggressive PA. Based on our results and combined with the experience of glioma immunotherapy, targeting macrophages/CD8+ TILs with anti-PD-L1 or other types of immune checkpoint inhibitors may provide a breakthrough in immunotherapy for aggressive PA. Whether immunotherapy based on TMZ treatment may benefit patients is expected to be confirmed by further preclinical and clinical trials.

## 5. Limitations

There were limitations to our study that should be acknowledged. First, the main limitation of this study is the low reproducibility of the immunohistochemical methods. However, immunohistochemistry is still the most important method for the diagnosis of PAs, based on the 2017 WHO classification of tumors of the pituitary gland [22]. Second, the conclusion that these data speak in favor of anti-lymphocytic or anti-macrophagic approaches in treatment is based on circumstantial evidence. Despite certain limitations of this retrospective study, the observed interesting clinical features have some enlightening significance and require further investigation.

## 6. Conclusions

Targeted therapy for immune cells could emerge as a potential treatment for aggressive PA. In PAs with a higher Ki-67 index, p53 expression, and more CD68+ macrophage infiltration, PD-L1 inhibitors may be more effective. In addition, the incomplete removal of the tumor, recurrent PA, and positive MMP-9 expression were confirmed as increased risk factors for recurrence of PA following surgery. Combined immunotherapy could open new chapters in the treatment of aggressive PA.

## Figures and Tables

**Table 1:** Characteristic clinical features of the patients with pituitary adenomas.

Characteristics	N (%)
Patients (*n*)	103
Age, mean years ± SD (range)	45.02 ± 14.91 (15–79)
Average follow-up time (range)	30.56 ± 6.53 (<1–35) months
Primary adenomas	79 (76.70%)
Recurent adenomas	24 (23.30%)
Gender	
Male	54 (52.43%)
Female	49 (47.57%)
Symptoms and signs	
Visual deficit	48 (46.60%)
Headache	45 (43.69%)
Menstrual disturbances	13 (12.62%)
Sexual dysfunction	6 (5.83%)
Polydipsia and polyuria	3 (2.91%)
Incidental finding	3 (2.91%)
Gross tumor volume median	7.08 cm^3^ (0.28 to 245 cm^3^)
Pituitary apoplexy	10 (9.71%)
Knosp classification grades	
Grade 0	43 (41.75%)
Grade 1	6 (5.83%)
Grades 2	10 (9.71%)
Grades 3	10 (9.71%)
Grades 4	34 (33.01%)
Pathological classifications	
Somatotroph adenomas	8 (7.77%)
Lactotroph adenomas	18 (17.48%)
Thyrotroph adenoma	1 (0.97%)
Corticotroph adenomas	5 (4.84%)
Gonadotroph adenoma	22 (21.36%)
Null cell adenoma	45 (43.69%)
Plurihormonal adenomas	4 (3.88%)
Secretory pituitary adenomas	54 (52.43%)
GH-secreting adenomas	4 (3.88%)
Prolactinomas	26 (25.24%)
ACTH-secreting adenomas	8 (3.88%)
FSH-secreting adenomas	2 (7.77%)
Plurihormonal-secreting adenomas	14 (13.59%)
Non-functional adenomas	49 (47.57%)
Immunohistochemical results	
Ki-67 median	1.50% (0–25%)
P53 positive	3 (2.91%)
MGMT expression	
<10%	4 (3.88%)
10–90%	50 (48.54%)
≥90%	49 (47.57%)
PD-L1 positive	19 (18.45%)
MMP-9 staining extent	
<5%	89 (86.41%)
5–25%	5 (4.85%)
26–50%	1 (0.97%)
>50%	8 (7.77%)
MMP-9 final scores	
negative	69 (66.99%)
weakly positive	24 (23.30%)
moderately positive	8 (7.77%)
strongly positive	2 (1.94%)
Surgical approach	
microscope transsphenoidal surgery	55 (53.40%)
endoscopic transsphenoidal surgery	27 (26.21%)
craniotomy	21 (20.39%)
Resection extent	
CR (complete resection)	55 (53.40%)
IR (incomplete resection)	48 (46.60%)

**Table 2:** Univariate and Multivariate Cox Regression Analysis for Survival.

Variables	Univariate Regression			Multivariate Regression		
	HR	95% CI	*p*	HR	95% CI	*p*
Pituitary apoplexy	1.291 × 10^−8^	0.00–Inf	1.00	8.43 × 10^−9^	0.00–Inf	1.00
Knosp grades	1.48	0.68–0.77	0.25	1.18	0.51–2.74	0.70
Recurrent tumor	3.64	0.51–25.82	0.20	2.14	0.26–17.20	0.47
Extent of resection	3.20	0.33–30.80	0.31	1.35	0.08–23.47	0.84
MMP-9 expression	4.22	0.44–40.61	0.21	4.80	0.38–60.65	0.23
MGMT expression	1.16	0.21–6.57	0.87	1.56	0.25–9.65	0.63
PD-L1 expression	2.91 × 10^−9^	0.00–Inf	1.00	4.22 × 10^−9^	0.00–Inf	1.00
Histopathological classifications	1.84	0.61–5.52	0.28	1.35	0.42–4.32	0.62
Functional classifications	0.94	0.58–1.52	0.80	0.82	0.49–1.39	0.46

**Table 3:** Univariate and Multivariate Cox Regression Analysis for Tumor Relapse.

Variables	Univariate Regression			Multivariate Regression		
	Exp(coef)	95% CI	*p*	Exp(coef)	95% CI	*p*
Pituitary apoplexy	1.27 × 10^−8^	0.00–Inf	1.00	3.24 × 10^−8^	0.00–Inf	1.00
Knosp grades	1.27	0.86–1.87	0.22	0.69	0.41–1.14	0.15
Recurrent tumor	5.21	1.40–19.41	0.01	5.13	1.07–24.61	0.04
Extent of resection	9.83	1.23–78.66	0.03	7.60	0.71–81.15	0.09
MMP-9 positive	4.18	1.11–17.88	0.04	4.40	0.91–21.21	0.06
MGMT expression	0.67	0.24–1.93	0.47	0.79	0.22–2.84	0.72
PD-L1 expression	0.57	0.12–2.73	0.48	1.10	0.13–9.33	0.93
Histopathological classifications	1.11	0.74–1.64	0.62	1.09	0.60–1.98	0.77
Functional classifications	0.98	0.70–1.36	0.89	0.94	0.61–1.44	0.76

## Data Availability

The clinical datasets generated during and/or analyzed during the current study are available from the corresponding authors on reasonable request.
